# Reconstruction of Multiple Facial Nerve Branches Using Skeletal Muscle-Derived Multipotent Stem Cell Sheet-Pellet Transplantation

**DOI:** 10.1371/journal.pone.0138371

**Published:** 2015-09-15

**Authors:** Kosuke Saito, Tetsuro Tamaki, Maki Hirata, Hiroyuki Hashimoto, Kenei Nakazato, Nobuyuki Nakajima, Akihito Kazuno, Akihiro Sakai, Masahiro Iida, Kenji Okami

**Affiliations:** 1 Department of Otolaryngology, Tokai University School of Medicine, 143 Shimokasuya, Isehara, Kanagawa, 259–1193, Japan; 2 Muscle Physiology & Cell Biology Unit, Tokai University School of Medicine, 143 Shimokasuya, Isehara, Kanagawa, 259–1193, Japan; 3 Department of Physiological Science, Tokai University School of Medicine, 143 Shimokasuya, Isehara, Kanagawa, 259–1193, Japan; 4 Department of Orthopedics, Tokai University School of Medicine, 143 Shimokasuya, Isehara, Kanagawa, 259–1193, Japan; 5 Department of General Thorathic Surgery, Tokai University School of Medicine, 143 Shimokasuya, Isehara, Kanagawa, 259–1193, Japan; 6 Department of Urology, Tokai University School of Medicine, 143 Shimokasuya, Isehara, Kanagawa, 259–1193, Japan; 7 Department of Gastroenterological Surgery, Tokai University School of Medicine, 143 Shimokasuya, Isehara, Kanagawa, 259–1193, Japan; University of Minnesota Medical School, UNITED STATES

## Abstract

Head and neck cancer is often diagnosed at advanced stages, and surgical resection with wide margins is generally indicated, despite this treatment being associated with poor postoperative quality of life (QOL). We have previously reported on the therapeutic effects of skeletal muscle-derived multipotent stem cells (Sk-MSCs), which exert reconstitution capacity for muscle-nerve-blood vessel units. Recently, we further developed a 3D patch-transplantation system using Sk-MSC sheet-pellets. The aim of this study is the application of the 3D Sk-MSC transplantation system to the reconstitution of facial complex nerve-vascular networks after severe damage. Mouse experiments were performed for histological analysis and rats were used for functional examinations. The Sk-MSC sheet-pellets were prepared from GFP-Tg mice and SD rats, and were transplanted into the facial resection model (ST). Culture medium was transplanted as a control (NT). In the mouse experiment, facial-nerve-palsy (FNP) scoring was performed weekly during the recovery period, and immunohistochemistry was used for the evaluation of histological recovery after 8 weeks. In rats, contractility of facial muscles was measured via electrical stimulation of facial nerves root, as the marker of total functional recovery at 8 weeks after transplantation. The ST-group showed significantly higher FNP (about three fold) scores when compared to the NT-group after 2–8 weeks. Similarly, significant functional recovery of whisker movement muscles was confirmed in the ST-group at 8 weeks after transplantation. In addition, engrafted GFP^+^ cells formed complex branches of nerve-vascular networks, with differentiation into Schwann cells and perineurial/endoneurial cells, as well as vascular endothelial and smooth muscle cells. Thus, Sk-MSC sheet-pellet transplantation is potentially useful for functional reconstitution therapy of large defects in facial nerve-vascular networks.

## Introduction

Cancer of the head and neck area are often diagnosed at advanced stages, and wide surgical resection is generally indicated for curative purpose. However, a wide range of nerve-vascular networks, including the facial nerves, tend to be removed with the lesion and this leads to various symptoms of nerve deficiency, such as difficulties in talking, eating or drinking, as well as drooling and muscle twitching, because the facial area includes special sense organs, sensitive muscular systems and various glandular organs; thus, damage reduces postoperative quality of life (QOL). In addition, facial neural networks are highly complex relative to other areas of the body.

In order to overcome these problems, various approaches have been attempted, and use of autologous nerve grafts has been the gold standard [1–3]. However, the number of suitable sites available for harvesting is limited, and the sacrifice of healthy function is inevitable. Therefore, application of artificial neural tubes has been attempted as an alternative treatment [1, 2], but the results remain unsatisfactory. Transplantation of stem cells, such as bone marrow mesenchymal stem cells [4, 5], adipose-derived stem cells [6, 7], Schwann-like mesenchymal stem cells [8], and dental pulp cells [9], has also been attempted with artificial neural tubes. However, the therapeutic effects are limited, because of difficulties in regenerating the complex networks of the facial nerve-vascular system in the large deficits with long gaps. In other words, it is difficult to bridge multiple nerve branches using nerve grafts or artificial conduits.

On the other hand, we have reported on the therapeutic effects of skeletal muscle-derived multipotent stem cells (Sk-MSCs), which have a synchronized reconstitution capacity for muscle-nerve-blood vessel units [10–12]. Recently, we further developed a three-dimensional (3D) patch transplantation system using gel-like Sk-MSC sheet-pellets [13] in order to improve handling, as they can be picked up with forceps and placed onto the desired site. These properties of Sk-MSCs sheet-pellet are considered to be suitable for the reconstitution of facial nerve-blood vessel networks.

Therefore, the purpose of this study is the application of our 3D transplantation system using Sk-MSCs sheet-pellets to the regeneration of facial nerve-vascular networks after severe surgical resection. Specifically, we examined whether sheet-pellet transplantation achieves the reconstitution of multiple nerve branches. For this purpose, we developed an animal model for large facial nerve-blood vessel network deficits, and a unique method of functional recovery measurement for the dominant muscles of whisker movement. Facial-nerve-palsy (FNP) was scored using a modification of Most’s method [14] during the recovery phase, and immunohistochemical detection of engrafted cells in vivo after transplantation was also performed.

## Materials and Methods

### Animals

In the present study, two sets of experiments were performed. The first set was performed in mice, and included histological and immunohistochemical analysis. Green fluorescent protein transgenic C57BL/6 mice (GFP-Tg mice; C57BL/6 TgN[act EGFP]Osb Y01, provided by Dr. M. Okabe [15], Osaka University, Osaka, Japan; age, 4–8 wk) were used as donors (n = 9, 5 males, 4 females), and wild-type C57BL/6N mice were used as recipients (n = 21, 8 males, 13 females). The second set was performed in rats. Sprague-Dawley (SD) rats were used for functional examinations (n = 12, 9 males, 3 females). Rat experiments included autologous transplantation, in which the soleus and gastrocnemius muscles of the right leg were removed and prepared for stem cell isolation. The animals were housed in standard cages and were provided food and water ad libitum under specific pathogen- and virus antigen-free condition in the independent and dedicated animal facility of our laboratory. The room temperature was kept at 23±1°C, and a 12:12-h light-dark cycle was maintained throughout the experiment. This study was carried out in strict accordance with the recommendations in the Guide for the Care and Use of Laboratory Animals of the National Institutes of Health. The protocol was approved by the Tokai University School of Medicine Committee on Animal Care and Use (Permit Number: 142032 for the rats, 142033 for the mice). All surgery was performed under sodium pentobarbital anesthesia or inhalation anesthesia (Isoflurane), and all efforts were made to minimize suffering.

### Sk-MSC isolation and preparation of stem cell sheet-pellets

**Fig 1:**
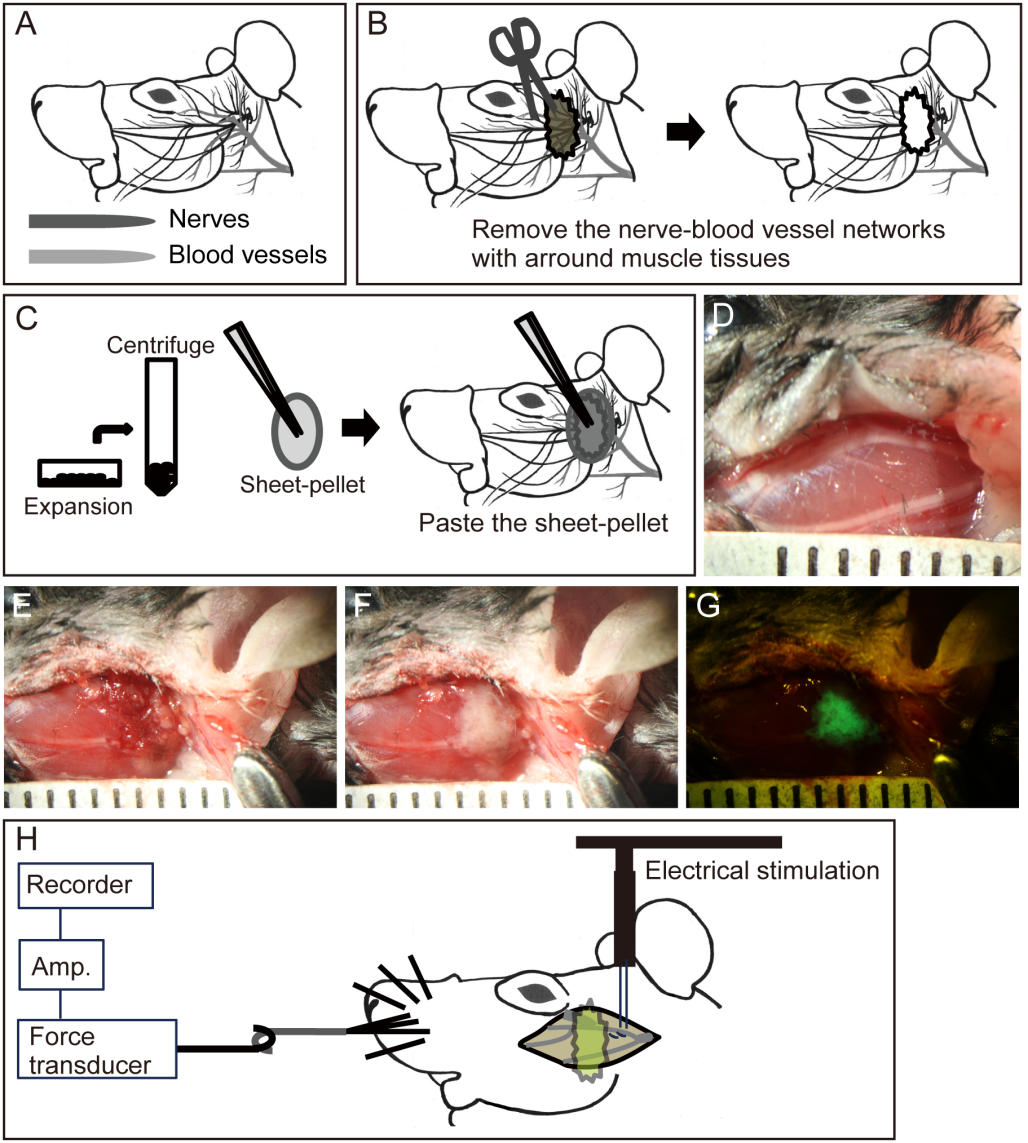
Summary of the facial nerve-blood vessel large deficit model, sheet-pellet transplantation, and functional measurement. (A-C) Schematic drawing and (D-G) stereomicroscopic photographs showing the large facial nerve-blood vessel network deficit model and sheet-pellet transplantation methods. Panel (A) shows the complex nerve-blood vessels networks in the face. (B) Large removal was performed near the convergent point of nerve-vascular networks. (C) Note that the sheet-pellets can be picked up with forceps. (D) Overview of surgical field. (E) Removal of tissues. (F) Patch transplantation of sheet-pellets. (G) Fluorescence microscopy of panel (F). Ruled markings indicate 1 mm. (H) Schematic drawing of contractility measurement in the whisker movement muscles. Amp = amplifier.

Sk-MSCs were enzymatically extracted from the thigh and lower leg muscles (tibialis anterior, extensor digitorum longus, soleus, plantaris, gastrocnemius and quadriceps femoris) of GFP-Tg mice, as described previously [10, 13, 16]. Stem cell sheet-pellets were also prepared as reported previously [13]. Briefly, whole non-minced muscles were treated with 0.1% collagenase type IA (Sigma-Aldrich, Tokyo, Japan) in Dulbecco’s modified Eagle’s medium (DMEM) containing 7.5% fetal calf serum (FCS) with gentle agitation for 30 min at 37°C, and were divided into fiber-bundles. Fiber-bundles were washed with Iscove’s modified Dulbecco’s medium (IMDM) containing 10% FCS, and were cultured in IMDM/20% FCS (100 units/ml penicillin G, 100 μg/ml streptomycin sulfate, and 10 μg/ml gentamycin sulfate and 0.1 mM β-mercaptoethanol) for 4 d. Cultures were then treated with trypsin-EDTA (0.05% trypsin, 0.53 mM EDTA; Life Technologies, Tokyo, Japan) and filtered through 70-, 40- and 20-μm nylon filters in order to remove muscle fibers and other debris, followed by washing and re-culture in IMDM/20% FCS for 3 d until confluence. After 7 d of expansion culture, confluent cells were gently detached from culture dishes with 2 mM EDTA solution, while maintaining as much cell-to-cell contact as possible (Fig 1C). Sheet-like cell aggregations were then collected in a centrifuge tube, and stem cell sheet-pellets were obtained after centrifugation (Fig 1C). In this process, 100~120 mg sheet-pellets were obtained, and 50~60 mg was applied in each experiment, with cell concentration being adjusted to the 1~1.2×10^5^/mg range.

For the rat autologous cell transplantation experiment, the right lower hindlimb soleus and gastrocnemius muscles were removed according to the method of the compensatory muscle hypertrophy model [17–19], and were prepared for the cell isolation and sheet-pellet method above. The rats were able to walk using the remaining plantaris muscle in this model.

Animal model for large facial nerve-blood vessel network deficit and sheet-pellet transplantation

Surgery and cell transplantation were performed unilaterally under inhalation anesthesia (Isoflurane; Abbott, Osaka, Japan). A schematic drawing and stereomicroscopic photographs of the procedure for preparing the model for the large facial nerve-blood vessel network deficit and sheet-pellet transplantation is shown in Fig 1. The networks of facial nerves and blood vessels are complex (Fig 1A and 1D). Facial skin insertion was performed only where needed for the operative field (refer to Fig 1D–1F), and wide-range removal of the facial nerve-blood vessel networks associate with the around muscle tissues (4×5 mm square in mice, and 8×10 mm in rats) were performed on the portion of multiple branches (including zygomatic and buccal branches) at 2 mm (mice) or 4 mm (rats) distal to the main facial nerve trunk (Fig 1B and 1E). Subsequently, eye, whisker and lip movement were significantly affected (wholly disappeared). Then, the prepared Sk-MSCs sheet-pellets were pasted onto the damaged area (Fig 1C, 1F and 1G). Transplanted sheet-pellets were covered with fibrin-gel (fibrinogen-based hemostatic agent; Beriplast, NIPRO, Osaka, Japan) in the ST group (n = 11, including 4 males). The same amount of culture medium and fibrin-gel (without stem cells) were transplanted in the control group (non-transplanted NT group, n = 10, including 4 males). For diminished eye closure movement in this animal model, eye moisture treatment was performed 3 times/day or more if necessary.

### FNP scoring

For the mouse model, facial-nerve-palsy (FNP) was scored based on modification of Most’s method [14]. Measurement was performed weekly throughout the recovery phase. We selected 3 categories based on the damage seen in the present model: eye closure; whisker reflection; and superior lip movement. Eye closure and lip movement were induced by gentle touching with soft paper to the lid margin of the eye and lower margin of the lip. Whisker movement was induced by gentle stimulation to the ipsilateral whiskers with a cotton swab. Reflections were recorded by video camera, and were repeatedly analyzed. Responses were independently graded at 0–4 levels when compared with the contralateral healthy side, as follows: 0 = no movement; 1 = little detectable movement; 2 = apparent movement; 3 = significant movement, but no symmetric movements; 4 = same as the contralateral healthy side with symmetric movements. The three scores were added, giving a total score of 0–12.

### Functional assessment of facial muscles

In this experiment, we used a rat model (n = 12, 9 males, 3 females), and contractility of dominant facial muscles were measured via electrical stimulation through the facial nerve root as an indicator of total functional recovery of networks. For this purpose, we developed a unique method of contraction measurement for the dominant muscles of whisker movement. A schematic drawing of this measurement is shown in Fig 1D. Measurements were performed *in situ* under sodium pentobarbital (40 mg/kg, with xylazine HCl 10 mg/kg, i.p.) anesthesia, and body (rectal) temperature was maintained at 36±1°C with a radiant heat source throughout the measurement. For this experiment, surgical removal of the right leg soleus and gastrocnemius muscles was performed for preparation of autologous Sk-MSC sheet-pellets 1 week before measurement under inhalation anesthesia. Measurements were then performed 3 times/rat (before surgery, 10 min after surgery and 8 weeks after surgery). First, the animal was fixed in a side lying position using surgical tape on a custom operating table that allowed stabilization of the head and limbs. After appropriate opening of the facial skin, the facial nerve root was carefully exposed, and surrounding tissues were coated with sterile mineral oil in order to prevent tissue drying and to minimize electric-noise interference. A bipolar silver (Ag/Ag) electrode (inter-electrode distance: 2 mm) was placed under the root of the facial nerve zygomatic branch. A stainless steel hook was tied off with 4–5 whiskers near the muffle using silk ligatures, and was attached to a force-distance transducer (FD-Pickup, TB-611T; Nihon Kohden, Tokyo, Japan) connected to a carrier amplifier (AP-621G; Nihon Kohden). Care was taken to avoid interference with the normal blood supply of the reference muscle, nerve and blood vessels. Twitches were then elicited using a single pulse electrical stimulation (1 ms duration, 0.5 Hz) via the zygomatic branch, at a voltage above the threshold for maximum response (1.5–4.0 V). Subsequently, peak tetanic tension was determined using stimulation frequencies of 10, 20, 40, 60, 80, 100 and 120 Hz of 0.5-s duration at 15-s intervals. The frequency that produced the highest tetanic tension was considered to be the optimal stimulation for tetanus. All mechanical and electrical measurements were recorded on a Linearcorder (Mark VII, WR3101; Graphtec, Tokyo, Japan) as raw analog data. The tetanic tension output was considered to be the total recovery of the facial nerve-muscle-blood vessel unit. After the first measurement, nerve network removal was performed, and at 10 min after surgery, the second measurement was performed to confirm functional reduction. Subsequently, sheet-pellets were transplanted and sutured. The third measurement was performed at 8 weeks after surgery. Recovery ratio was determined based on the pre-surgery values, and the results were compared between the ST (n = 6) and NT (n = 6) groups, which were randomly selected.

### Macroscopic Observation and Immunostaining

**Fig 2:**
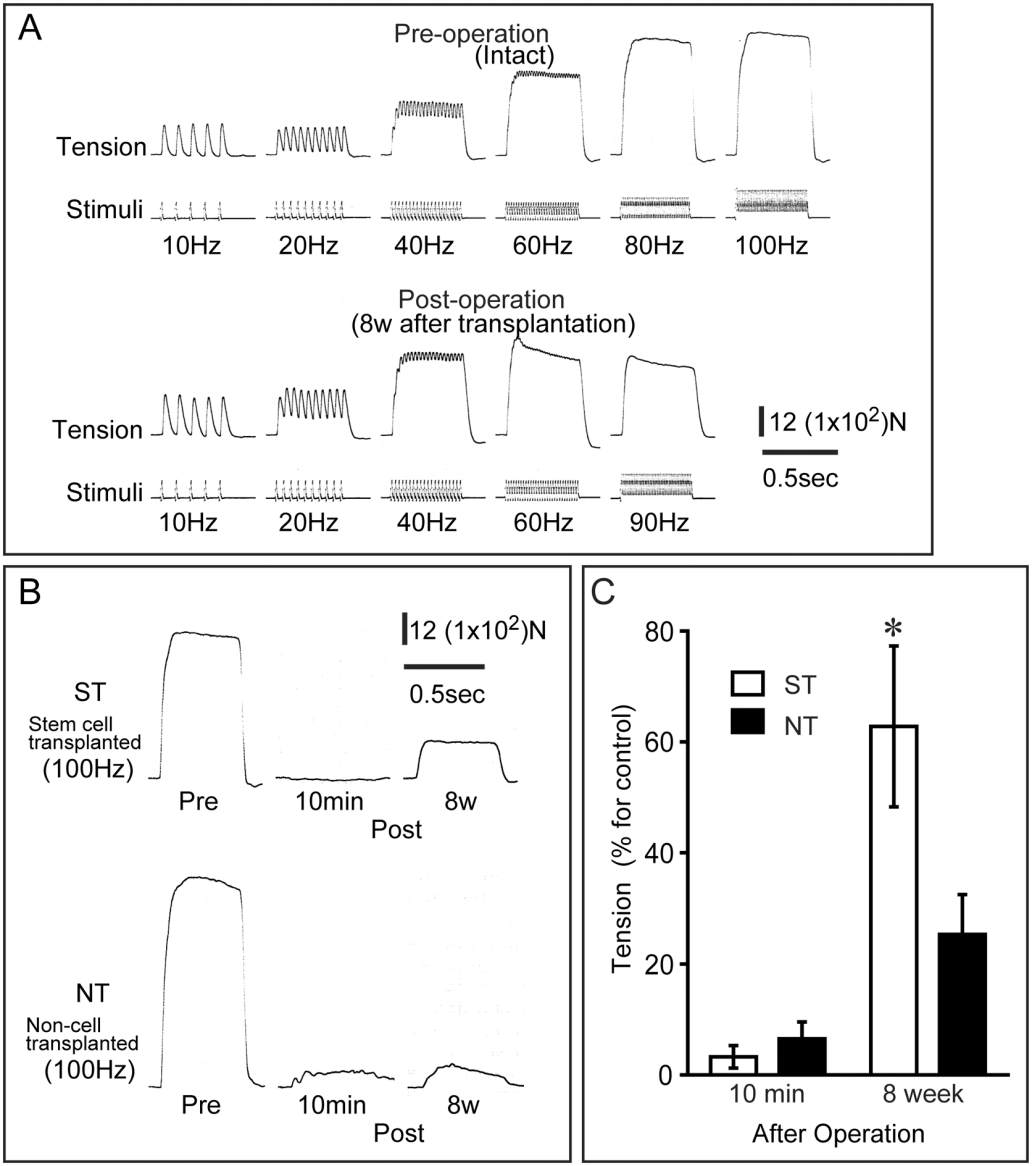
Evaluation and comparison of functional recovery in rat model. (A) Tetanic tension development following increase in stimulation frequency Pre- and 8weeks Post-operation. (B) Typical individual measurements of maximum tetanic tensions Pre, Post-10 min and 8weeks in the ST and NT rats. (C) Recovery of tetanic contractions in whisker movement muscles at 10 minutes after tissue removal and at 8 weeks after transplantation. Values are expressed in terms of % vs. contralateral side. Note that tetanic tension output was wholly absent at 10 min after removal (B and C). *P< 0.05.

At 8 weeks after transplantation, recipient mice were given an overdose of pentobarbital (60 mg/kg, + xylazine HCl 10 mg/kg, i.p.), and engraftment of donor-derived GFP^+^ cells into the damaged portion was confirmed by fluorescence stereomicroscopy (SZX12; Olympus, Tokyo, Japan, Fig 2). Recipient mice were then perfused with warm 0.01 M phosphate buffered saline (PBS) through the left ventricle, followed by fixation with 4% paraformaldehyde/0.1 M phosphate buffer (4% PFA/PB). Surgically excised tissues were removed and post-fixed overnight in 4% PFA/PB, washed with graded sucrose (0–25%)/0.01 M PBS series, and quick frozen by isopentane pre-cooled by liquid nitrogen, followed by storage at -80°C. Subsequently, 7-μm cross-sections were obtained. Skeletal muscle fibers were stained with rabbit polyclonal anti-skeletal muscle actin (αSkMA; dilution = 1:200; incubation = room temperature for 2 h; Abcam, Cambridge, UK). Localization of nerve fibers (axons) was confirmed by rabbit polyclonal anti-Neurofilament 200 (N-200, 1:1000; room temperature for 1 h; Sigma, St. Louis, MO). Schwann cells were detected by anti-p75 (rabbit polyclonal, 1:400, 4°C overnight; CST, Boston, MA) and rabbit anti-GULT-1 (1:100, room temperature for 1 h; Diagnostic BioSystems, Pleasanton, CA) was used for identification of perineurium. Myelin formation was detected by anti-myelin basic protein (MBP; rabbit polyclonal, dilution = 1:200; incubation = room temperature for 2 h; MILLIPORE, Billerica, MA). Neuromuscular junctions were detected by α-bungarotoxin (Alexa Fluor 594 conjugated, 1:100, room temperature for 1 h; Molecular Probes, Eugene, OR). Blood vessels were detected with rat anti-mouse CD31 (1:500, 4°C overnight; BD Pharmingen, San Diego, CA) monoclonal antibody and mouse monoclonal α-smooth muscle actin (αSMA, Cy3-conjugated; 1:1500; room temperature for 1 h; Sigma). Reactions were visualized using Alexa Fluor-594-conjugated goat anti-rabbit and anti-rat antibodies (1:500, room temperature for 2 h; Molecular Probes, Eugene, OR). Nuclei were counter-stained with DAPI (4,6-diamino-2-phenylindole).

### Statistics

Differences between two groups were tested by Student’s t test, and the level of significance was set at p<0.05. Values are expressed as means ± SE.

## Results

### Functional recovery

In the measurement of tetanic tension output of whisker movement muscles in the rat model, typical raw data of pre and post operation was shown in Fig 2A. In the present study, tension measurement was done through the whisker bundle, but the records showed quite similar to the textbook tension responses, as twitches, incomplete/complete tetanus, following gradual increases in stimulation frequency, when directly connected to the muscle tendon, both in pre and post operation (Fig 2A). Therefore, we decided that the present method was available as for the functional examination/comparison. Typical tetanic tensions of pre, and post 10 min and 8w of both ST and NT group are also shown in Fig 2B. Tension output was wholly disappeared at 10 min post operation in both groups. At 8 weeks post-operation, however, a complete tetanus was obtained in the ST group, although the value was still lower than the pre-value, but the NT group showed incomplete tetanus even in the 100Hz stimulation (Fig 2B). These post-operative results were averaged between two groups, as the percentages of the pre values, and significant reductions in tetanic tension (below 10%) were confirmed at 10 min after surgical resection both in ST and NT groups (Fig 2C). This indicates that the nerve network upstream of the whisker movement muscles was removed by surgery. However, at 8 wk after sheet-pellet transplantation, a significantly higher recovery ratio was also confirmed in the ST-group (over 60%), while the NT-group showed 25% (Fig 2C).

**Fig 3:**
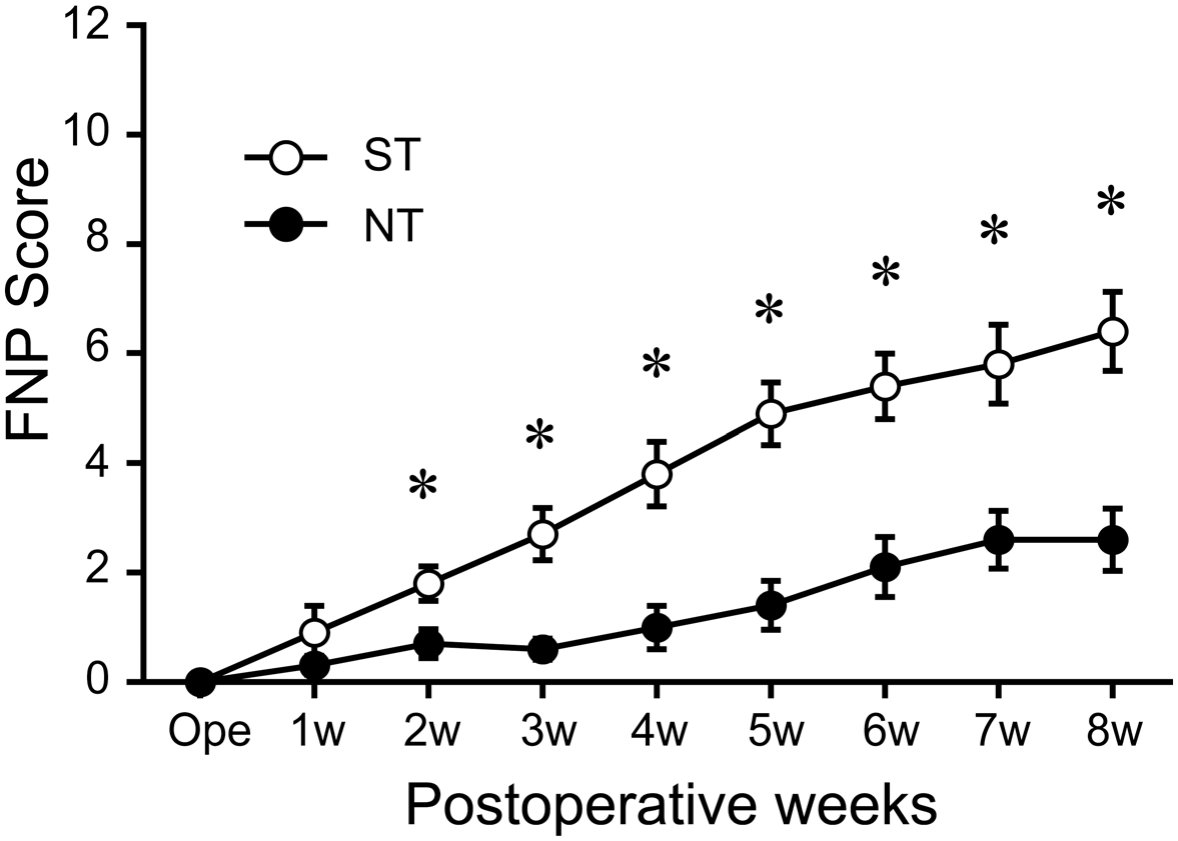
FNP scores during 8-week recovery phase in mouse experiment. Significant differences are detected after 2 weeks of recovery phase. *P< 0.05.

Similarly, at 2wk after transplantation, the ST-group showed a significantly higher FNP score (about 3 times) than the NT-group, and this trend was maintained through the recovery phase (Fig 3). At 8 wk after transplantation, in the ST-group, FNP score on the treated side was over 50% of that on the contralateral side, whereas the NT-group showed a score of 15% (Fig 3). Thus, favorable reconstitution of eye and whisker movement-related tissues, particularly nerve networks, was observed in the mouse experiment. These results in the rat and mouse experiments were similar, although the former assessed electrically induced in situ tetanic tension and the latter assessed voluntary movement.

### Immunohistochemical evaluation of engrafted cells

**Fig 4:**
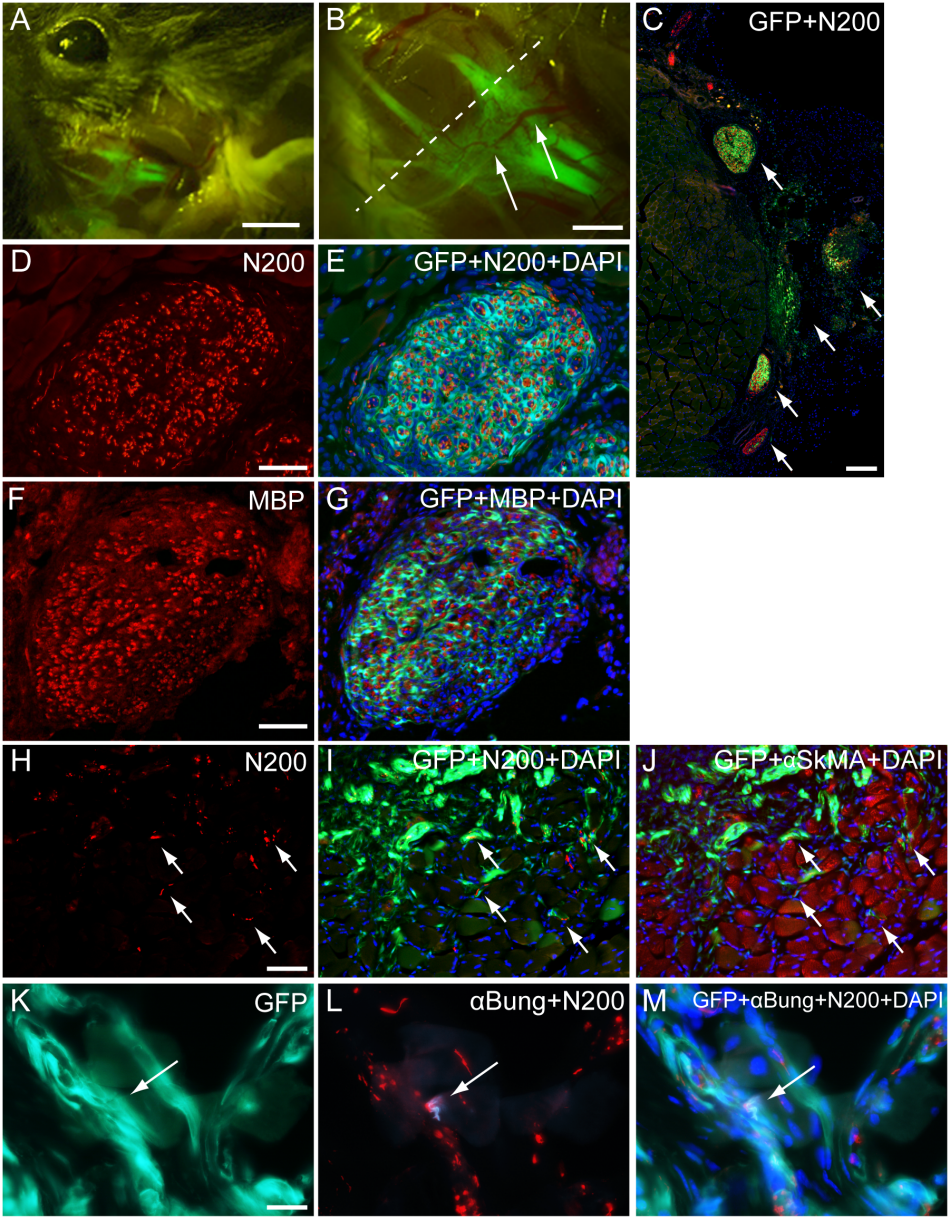
Detection of engrafted GFP^+^ tissue and cells in mouse experiment. (A, B) Macroscopic detection of multiple branches of facial nerves under fluorescence stereomicroscopy. GFP^+^ nerve branches in multiple directions were evident, and several vascular branches were also observed on the surface of GFP^+^ nerves (arrows). (C) Histological profile taken from the dotted line in panel B. Five aggregated conduit nerves were detected (arrows). (D and E) Axon staining by N200. GFP^+^ tissues encircled individual and/or several axons (E, merge). (F and G) myelin staining by MBP. Re-myelination was also established. (H-J) GFP^+^ tissues/cells show close relationships with N200^+^ axons (arrows in F and G) and were present in the SkMA^+^ muscle fiber area (arrows in H). (K-M) GFP^+^ tissue/cells can be seen close to the neuromuscular junction, as detected by αBungarotoxin (arrow in K and L), showing extension into the motor nerve end (arrow in M). Bars in A = 3 mm; B = 1 mm; C = 200 μm; D-E, F-H = 50 μm; and I-K = 20 μm.

**Fig 5:**
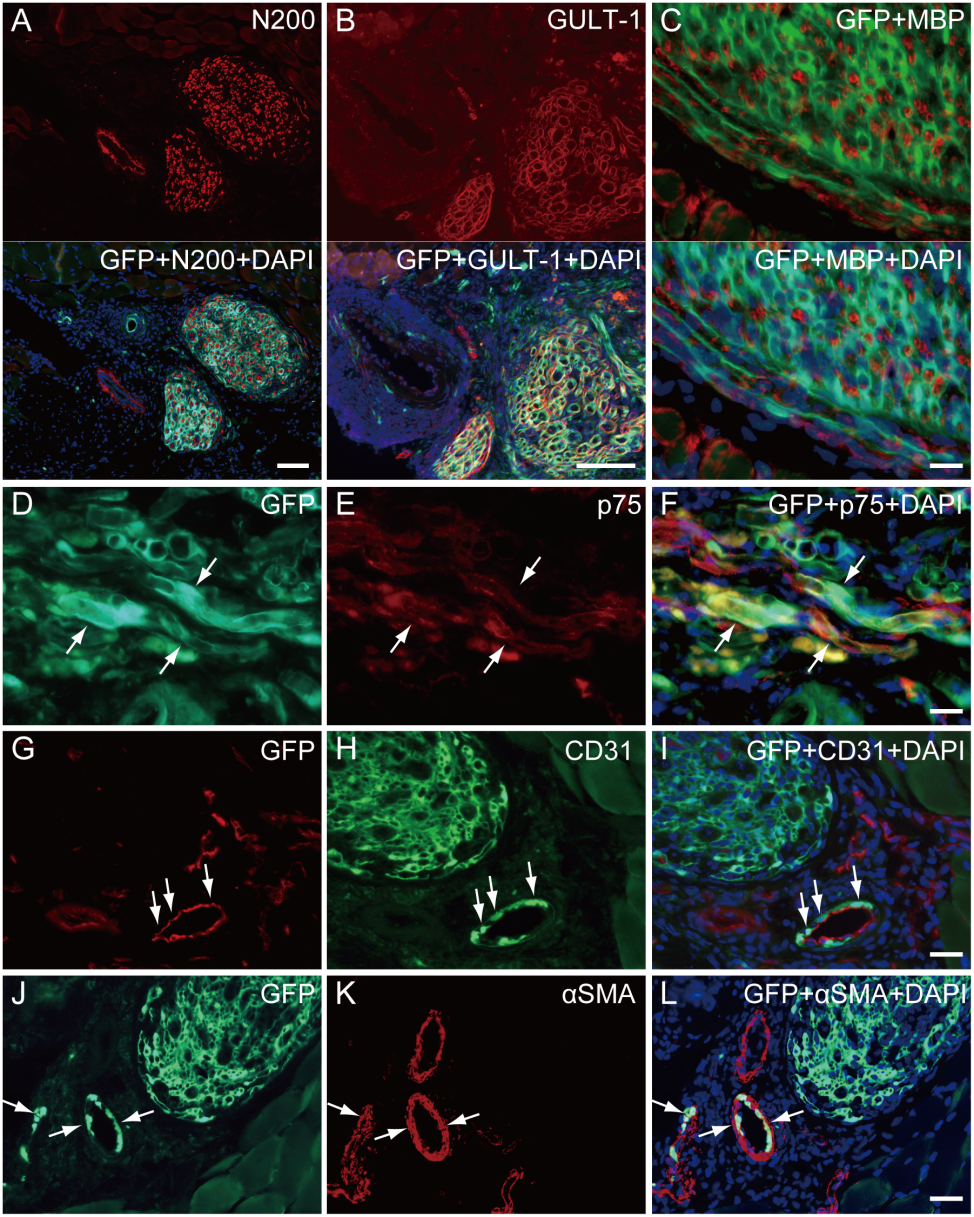
Detailed contributions of engrafted GFP^+^ cells in mouse experiment. (A) Axon staining by N200. (B) Perineurium/endoneurium staining by GULT-1. (C) Myelin staining by MBP. Regenerated axons and myelin were encircled by GFP^+^ tissues, which were positive for the perineurium marker GULT-1. (E) Schwann cell staining by p75. (H) Vascular endothelial cell staining by CD31. (K) Vascular smooth muscle staining by αSMA. Panels (F, I, L) show merged view of each staining. Engrafted GFP^+^ cells also differentiate into Schwann cells (arrows in D-F), vascular endothelial cells (arrows in G-I) and smooth muscle cells (arrows in J-L). Bars in A and B = 100 μm; C, D-F, G-I, J-L = 20 μm.

On in situ fluorescence macroscopic observation, a large volume of GFP^+^ tissues was engrafted in the damaged portion at 8 wks after transplantation (Fig 4A). They showed dendritic shape, which is typical for a neural network (Fig 4B). Vascular networks were also detected around GFP^+^ dendritic tissues (arrows in Fig 4B). When histological sections (cross-section) obtained from the dotted line in panel B were stained with N200 (axon staining), multiple nerve thick nerve stumps including GFP^+^ tissues could also be seen (Fig 4C). These data indicated the reconstitution of multiple, thick nerve branches in the facial nerve networks after sheet-pellet transplantation. In addition, each nerve stump included GFP^+^ tissues encircling N200^+^ axons, and some of these wholly comprised GFP^+^ tissues, thus showing a close relationship with nerve fibers (Fig 4D and 4E). Similarly, re-myelination of GFP^+^ cell engrafted nerve was also confirmed by the MBP staining (Fig 4F and 4G). This relationship between GFP^+^ cells and nerve networks was further maintained between skeletal muscle fibers (Fig 4H–4J), and reached the neuromuscular junction (Fig 4K–4M). Taken together, these results suggest that GFP^+^ engrafted cells migrated and contributed to nerve fiber regeneration from the conduit portion to the motor nerve endplate. Detailed contributions of engrafted GFP^+^ cells are shown in Fig 4. On serial sections of N200^+^ axons encircling GFP^+^ tissues (Fig 5A), the GFP^+^ circles were positive for GULT-1 (Fig 5B), and were thus considered to be perineurium. Furthermore, a higher magnification photograph with anti-MBP staining clearly showed that GFP^+^ tissues encircled one or multiple myelinated axons (Fig 5C), suggesting they were endoneurium/perineurium. Engrafted GFP^+^ cells also showed positivity for p75 (Fig 5D–5F), which is indicative of Schwann cells. Differentiation into vascular cells, such as CD31^+^ endothelial cells (Fig 5G–5I, arrows) and vascular smooth muscle cells (Fig 5J–5L, arrows), was also confirmed.

## Discussion

Although the present study focuses on a therapeutic stem cell transplantation method, we did not use any tube materials, in contrast to most previous studies, which have combined the use of a tube conduit and stem cells [5, 7, 9, 20]. In addition, the present results clearly indicate that the transplanted Sk-MSC sheet-pellets contributed to the reconstitution of multiple branches of disrupted facial nerves following cell differentiation into all peripheral nerve supportive cells, such as Schwann cells and perineurial/endoneurial cells (Figs 4 and 5). This nerve branch formation after stem cell transplantation has never been reported in the facial nervous system, and is considered to be typical of the present Sk-MSC sheet-pellet transplantation method. Therefore, it is possible that the engrafted Sk-MSCs were more strongly influenced by disrupted nerve stumps than by other tissues, probably as a result of neurotropic and/or neurotrophic factors between the proximal and distal nerve stumps. The potential for perineurium/endoneurium formation during early stages of nerve regeneration, which is a typical characteristic of Sk-MSCs [10–12], also contributed to the concentration of these factors and the determination of nerve growth direction/extension.

The other important factor in successful reconstitution is related to the characteristics of the sheet-pellets [13]. The manner in which stem cells are delivered to the injury site must be considered. Nerve regeneration is possible with: 1) Direct microinjection, but this is only available when outermost layer (epineurium) is preserved; 2) Live nerve grafts, which are the present gold standard [21]; 3) Suspension within artificial tubes, which is the most general approach [5, 7, 9, 20], but is not suitable for multi-branch nerve reconstitution; 4) Direct seeding over the wound area, which may be applicable to multi-branch reconstitution, but the prevention of cell diffusion after transplantation is problematic. The present sheet-pellet preparation overcame this problem by maintaining the cell-to-cell contact relationship [13].

The initial distance of nerve damage in this model was about 4 mm in mice at surgery (see Fig 1E), and sheet-pellets were simply placed to cover the wound area (Fig 1F and 1G). However, the GFP^+^ recovered nerve branches were clearly longer than 4 mm (some reached 7–8 mm total; Fig 4A and 4B). This suggests that the engrafted Sk-MSCs bridged the disrupted nerve branches, and then migrated toward non-damaged nerve portions in both the proximal and distal directions. Therefore, this migration activity may also be the main contributor to multi-directed nerve reconstitution. Similar migration of Sk-MSCs was also observed in bridging therapy for the long-gap sciatic nerve deficient model [16]. In particular, this migration capacity was stronger in the distal direction, and GFP^+^ cells could be seen up to the motor nerve ends (Fig 4K–4M). Thus, the engrafted Sk-MSCs likely to further contributed to functional re-connections to the skeletal muscle fibers. Consequently, the multiple nerve branch reconstitution resulted in favorable recovery of eye, whisker and lip movement in vivo, and over 60% recovery of whisker movement dominant muscle contractions in situ within 8 weeks (Figs 2 and 3). To our knowledge, this is the first report on statistical and objective measurement of facial nerve functional recovery after stem cell transplantation, although EMG measurements have been reported [7, 8, 22]. Irrespective, over 60% functional recovery represents a substantial therapeutic effect in the treatment of severely damaged facial nerves.

The acceleration of blood vessel formation by Sk-MSCs was also observed with differentiation into vascular endothelial and smooth muscle cells (Figs 4 and 5). This ability was also exerted in various tissues [10, 23–25], and the favorable supply of O_2_/nutrition with the elimination of CO_2_ and various waste products in and around the damaged tissue area was expected. Thus, this assisted revascularization may also act as an accelerator of tissue regeneration.

Stem cell transplantation therapy is an attractive alternative to nerve autografts, and several types of stem cell have been studied, including bone marrow mesenchymal stem cells [5], adipose-derived stem cells [7], Schwann-like mesenchymal stem cells [8] and dental pulp cells [9], with artificial neural tubes. However, the extent of transplanted cell survival and differentiation has not been sufficiently elucidated, while an improvement in regeneration outcomes also has been observed in the absence of transplanted cells [20]. These results suggest that stem cell transplantation should be performed with adjuvant therapy comprising neurotropic and neurotrophic factors associated with revascularization. In particular, the delivery of NGF [26], BDNF [27], GDNF [28], FGF-2 [29] and CNTF [30] have positive effects on nerve regeneration, and Ninjurin [31] and Galectin [32] promote axonal growth. Similarly, VGEF [33] and HGF [34] are important for blood vessel formation. In these regards, sufficient expression of these factors in Sk-MSCs sheet-pellets prior to transplantation was confirmed [16]. Therefore, adjuvant therapy with the aim of promoting paracrine effects on both recipient and engrafted cells after transplantation, may also be possible, as was demonstrated in the therapeutic use of long-gap sciatic nerve transection [16].

Concerning to the misdirection of facial nerve regeneration, which causes synkinesis [35]; we considered that complete prevention of misdirection is quite difficult and may be impossible in any methods including the present case. However, as mentioned above, re-connection of transected nerve in the present sheet-pellet transplantation method is considered to be standing largely depend on the facilitated formation of the perineurium/endoneurium during early stages, which is the typical characteristics of Sk-MSCs transplantation [10, 11, 16]. They possibly contribute to condense nerve growth, neurotropic and neurotrophic factors between the proximal and distal nerve stumps, and activated Schwann cells, which in both donor- and recipient-derived, regulate axonal extension in their circle toward the target transected stamps. These encircling features of Sk-MSCs can be confirmed consistently in Figs 4 and 5. This physiological nerve-guide process is similar to the case of embryonic peripheral nerve development, such as the interplay between the axonal growth cone and environmental guidance cues [36, 37]. In our unpublished data, functional recovery of the downstream muscle tension already observed at 10 days after human skeletal muscle-derived stem cell transplantation. Therefore, we believe that early commencement of rehabilitation, such as the biofeedback method using a mirror [38], may be possible and necessary for the clinical application, and selective neuronal activation (increased physiological demands) in the early recovery phase may work benefit to decrease misdirection of axonal regeneration.

## Conclusion

The present Sk-MSC sheet-pellet transplantation method showed active contributions to the reconstitution of multiple branches of facial nerves and blood vessels, following cellular differentiation into Schwann cells and perineurial/endoneurial cells, as well as vascular endothelial cells, smooth muscle cells and pericytes. Furthermore, these contributions induced 50–60% functional recovery within 8 weeks. These results suggest that the present method is a potentially useful tool for the recovery of various facial functions in large nerve-blood vessel network deficits following tumor removal or other traumatic events.

## Supporting Information

S1 ARRIVE Checklist ARRIVE Guidelines Checklist. (PDF)
